# [2.2]Paracyclophane Materials – Status and Perspectives

**DOI:** 10.1002/marc.202500145

**Published:** 2025-04-28

**Authors:** Henrik Tappert, Stefan Bräse

**Affiliations:** ^1^ Karlsruhe Institute of Technology (KIT) Kaiserstraße 12 76131 Karlsruhe Germany

**Keywords:** [2.2]paracyclophanes, advanced materials, chemical vapor deposition, chiral polymers, conjugated polymers, metal‐organic frameworks

## Abstract

[2.2]Paracyclophane (PCP) has emerged as a versatile building block in polymer science owing to its unique bicyclic structure, rigidity, and inherent chirality. To promote this to the broader public and highlight its versatility, this paper provides a concise overview of recent advancements in PCP‐based polymers, including their synthesis, structural features, and diverse applications. Functionalized PCP side chain and backbone polymers exhibit remarkable properties, including π‐stacking, tunable optoelectronic activity, and an easily defined 3D structure. Applications span from advanced coatings using chemical vapor deposition to semiconductors and emitters, as well as chiral materials for sensing and catalysis. Additionally, the incorporation of PCP into metal‐organic frameworks opens up new avenues in materials science. These findings highlight the PCP's potential to drive innovative macromolecule engineering and technology solutions.

## Graphical abstract

[2.2]Paracyclophane (PCP), with its rigid and chiral structure, drives advancements in polymer science. This paper highlights innovative PCP‐based materials, including functional side chain and backbone polymers, chemical vapor deposition coatings, and metal‐ organic frameworks. It explores how these materials are applied in sensing, gas separation, or optoelectronics, underlining the molecule's versatility in macromolecular engineering.

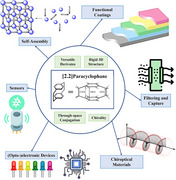

## 1 Introduction

The world of macromolecules is a continuously expanding domain that occupies a significant portion of chemical space and has applications across various fields, including biology, medicine, chemistry, catalysis, engineering, and industrial processes. While many molecular base motifs, such as styrene and methacrylate, are well‐known and extensively studied in polymer science, this publication highlights a less familiar but equally fascinating molecule: [2.2]paracyclophane (PCP).

**Figure 1:**
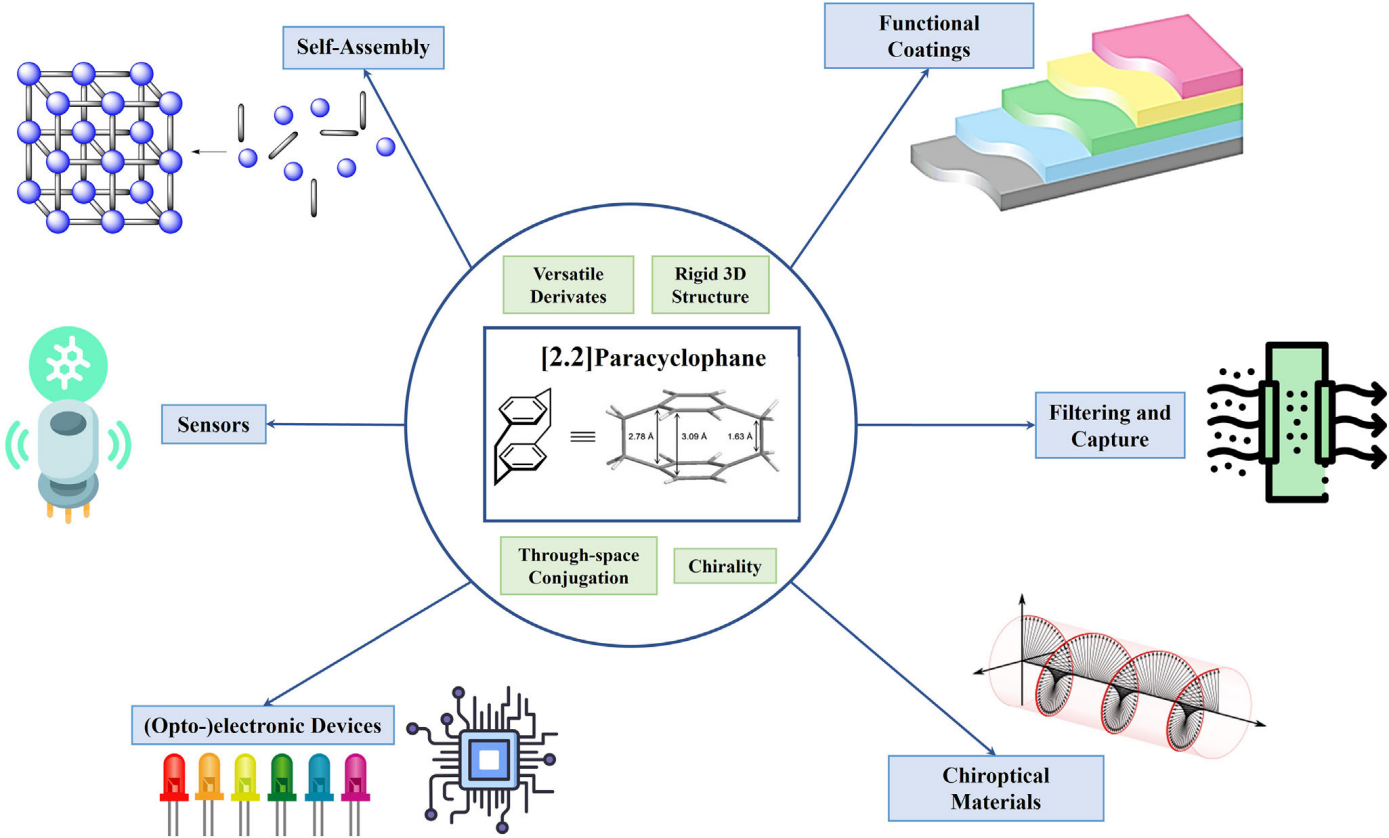
Overview of structure, properties, and applications of PCP.

This unique compound, frequently employed by research groups at the Karlsruhe Institute of Technology (KIT), demonstrates remarkable versatility in both synthesis and application.^[^ ^1^, ^2^ ^]^ At the core of its utility lies its distinctive structure, which consists of two tightly stacked co‐facial benzene rings connected by two ethylene bridges at their *para* positions (**Figure** **1**).

The architecture of [2.2]paracyclophanes imposes a rigid, locked conformation that distinguishes it from cyclophanes with longer, more flexible bridges. The short ethylene linkers (2.78 Å) restrict the rotation of the rings, forcing them into a non‐planar, bent configuration. The maximum separation between the benzene rings is only 3.09 Å, which is significantly shorter than the interlayer spacing in unstrained systems, such as graphite (3.40 Å). The short interlayer distance leads to strong transannular *π*–*π* interactions, which significantly impact its reactivity and optoelectronic behavior. The pronounced strain between the benzene rings results in high ring strain energy and a modified electronic structure, manifesting as distinct shifts in the UV–vis absorption spectra. Furthermore, the strain enhances the molecule's chemical reactivity, making it more prone to reactions that occur less frequently in unstrained aromatic systems, such as electrophilic substitution.

**Figure 2:**
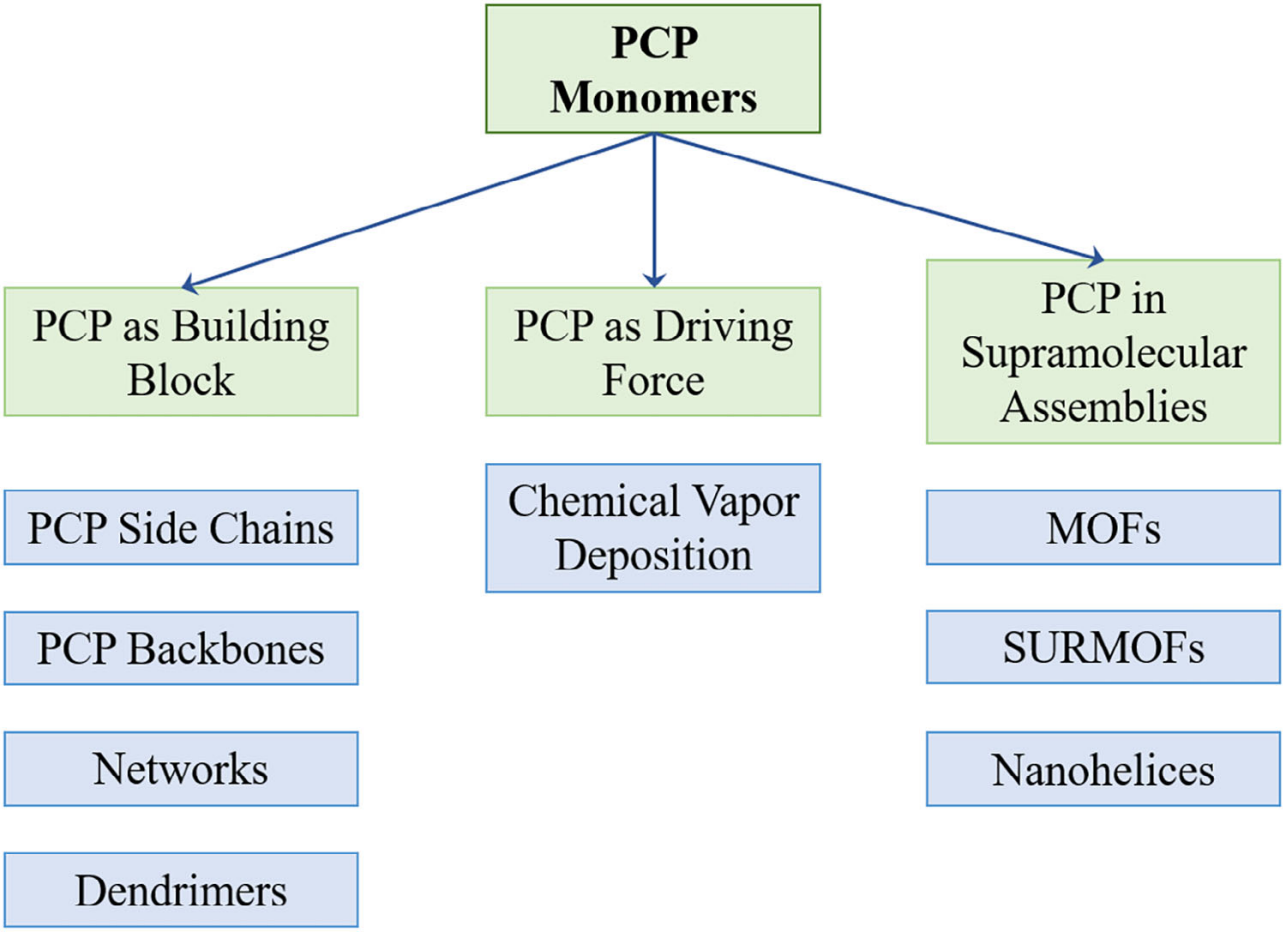
An overview of the possible macroscopic structures using PCPs is discussed below.

Additionally, the rigid spatial arrangement of the benzene rings in PCPs gives rise to planar chirality when the molecule is appropriately substituted, further expanding its versatility in chemical applications. Therefore, since its discovery in 1949, PCP and its derivatives have been successfully utilized in numerous fields such as catalysis,^[^ ^3^ ^]^ (opto‐)electronics,^[^ ^4^, ^5^ ^]^ or even biological systems.^[^ ^6^ ^]^ Many of the intrinsic properties of PCP, including rigidity, chirality, and a through‐space conductive system, make it an ideal candidate for both small molecule applications and the construction of a wide variety of functional macroscopic structures (**Figure** **2**).

This paper aims to showcase the nearly untapped potential of PCP as a monomer for advanced polymer synthesis to a broader audience. We explore various monomer variations, polymerization methods, and innovative applications, highlighting the significance of [2.2]paracyclophane as a building block in advancing polymer science and engineering in the last two decades.

## 2 Polymers Based on the [2.2]Paracyclophane Motif

### 2.1 PCP as a Side Chain Modification of Polymers

**Figure 3:**
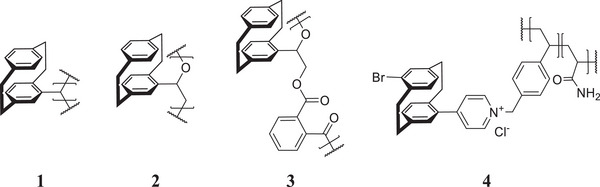
Selection of literature‐known polymers with PCP side chains.^[^ ^7^, ^8^, ^9^ ^]^

The versatility of the PCP core enables plenty of ways to achieve polymers with various functions and 3D structures. One of the simplest ways to achieve PCP polymers is functionalizing a PCP monomer with groups that can be used in established polymerizations to build various well‐known backbones bearing PCP as the side chain. This enables the incorporation of a wide range of polymerization techniques while introducing new functionalities derived from the PCP motif (**Figure** **3**). Particularly intriguing is that the intrinsic planar chirality of substituted PCPs can be transferred to achieve chiral polymers, which were successfully implemented in multiple instances. However, examples remain rare in the literature due to the challenges associated with polymerizing bulky side chains, which often necessitate the use of co‐monomers or spacer groups between the PCP and the backbone.

For example, Wada et al. employed a BF_3_‐catalyzed polymerization of chiral diazomethyl PCP to produce poly(methyl‐PCP) (**1**) enantiomers (Figure 3), which exhibit clear mirror behavior in CD spectra with a strong cotton effect.^[^ ^9^ ^]^ The proximity of the rigid and sterically demanding side chain results in a very stable, π‐stacked polymer, even compared to similar PCP polymers, as observed in its thermogravimetric analysis (≈50 °C increase) and absorption spectra. However, polymerization suffered from low yields of up to 20% and a short chain length of up to 2000 g mol^−1^. Both can be attributed to the steric hindrance of the side chain. They do not follow up with an application but propose chiral TADF emitters.

Similarly, Kern et al. synthesized a PCP oxirane that exhibits central and planar chirality and used polymerization with an aluminum‐based catalyst and an alkylammonium to produce chiral polyethers **2**, one of the most widely produced polymer classes (Figure 3).^[^ ^7^ ^]^ Additionally, they employ copolymerization with phthalic anhydride to expand their scope to polyesters **3**. All polymers exhibit optical activity of up to 46°mL g^−1^ dm^−1^, demonstrating their chirality and thus their potential use in fields such as catalysis or sensing. The main challenge of this monomer is its instability toward ring opening, especially in acidic conditions, which limits yields and chain length to below 20% and 1900 g mol^−1^, respectively. Further research and process optimization would be required for upscaling and practical applications.

**Figure 4:**
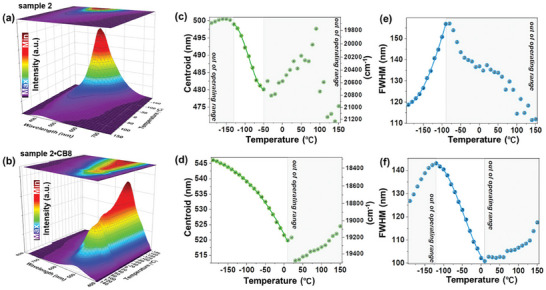
a, b) The emission spectra of selected samples as a function of temperature. c, d) The determined λ_centroid_ of the emission band and the determined FWHM of the band as a function of temperature. Reproduced under the terms of the CC‐BY license. 2024, published by Wiley‐VCH GmbH.^[^ ^8^ ^]^

When looking for a more practical application of this class of PCP polymers, Qiu et al. provide a good example of how to utilize properties known for a PCP monomer and improve upon them by scaling up to the macroscopic level.^[^ ^8^, ^10^ ^]^ They utilized the PCP side chain to create temperature‐sensitive sensors with a range of −190 to 140 °C, exhibiting great sensitivity, especially in the cryogenic region (**Figure** **4**).^[^ ^8^ ^]^ They equipped pyridyl PCP with vinylbenzyl groups and copolymerized it with acrylamide using azobisisobutyronitrile (**4**, Figure 3). This allows the combination of the sensor abilities of the PCP moiety with good water solubility. To further enhance the luminescence, the side chain is also complexed with cucurbit[8]uril (CB8) in a guest‐host system, which is an established interaction for PCP.^[^ ^10^ ^]^

The synthesis of polymers with PCP side chains can be further applied to various other functional groups, ranging from common ones like vinyl PCP to more unusual ones, such as diazoacetyl‐4‐methyl‐PCP.^[^ ^11^ ^]^ This area, like much of the work involving PCP, remains an active field of research at KIT and globally.

While PCP‐functionalized polymers present exciting opportunities, several challenges remain. For one, the price of PCP is relatively expensive when compared to common industrial monomers. This is partly due to PCP being a niche product, only produced and sold on a laboratory scale with less optimized methods, which is even more true for derivatives and chiral variations. This makes PCPs mainly interesting for highly specialized applications rather than as bulk material. This is reflected in the tested and proposed applications found throughout the discussed literature involving sensors, electronics, or biomedical materials. A similar issue is the lack of fundamental research on the reactivity of PCPs, resulting in inconsistent availability of established routes for many derivatives compared to other aromatic compounds. However, this is to be expected for such a young field and will likely improve over time. Another caveat in the application of PCP monomers is their thermal degradation, which begins at ≈230 °C. At this temperature, the ethylene bridges open, breaking the PCP. While this rules out many polymers incorporating PCPs for high‐temperature applications, it also presents an opportunity for further chemistry. A polymerization method based on this principle will be discussed in Chapter 3.

### 2.2 PCP as Backbone

**Figure 5:**
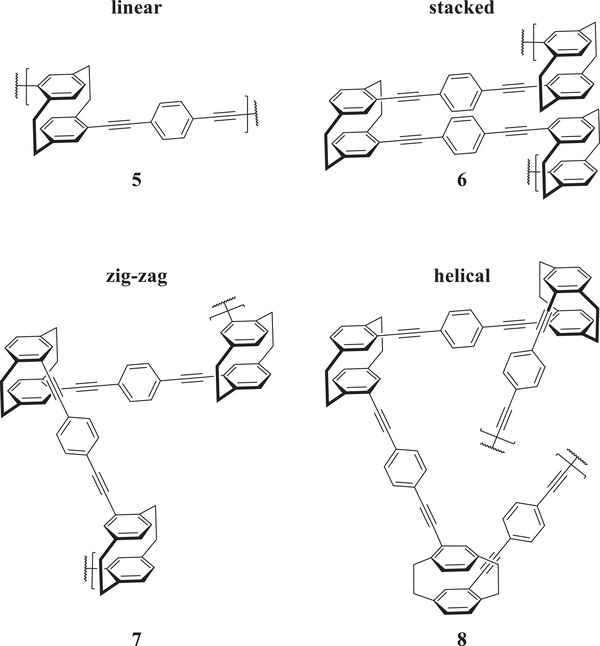
3D structuring through variation of PCP isomer.^[^ ^12^ ^]^

Aside from its use as a side chain, incorporating PCP into the backbone is also possible, for example, by using step‐growth techniques such as cross‐coupling polymerization. The rigid arrangement of PCP and its substituents in space enables controlled structuring in three dimensions, which is impossible with similar but planar motifs, such as benzene. The defining elements of the 3D structure, such as its angles, can be controlled by choice of stereoisomer. At the same time, the expansion and pocket sizes can be varied by adding spacing groups, such as alkyne or phenyl.^[^ ^12^ ^]^ Aside from linear polymers arising from *para*‐ and pseudo‐*para*‐substituted PCP **5**, it is possible to form a rigid sheet of the stacked polymer **6** by using pseudo‐*geminal* PCP, which reverses direction at every PCP moiety. Furthermore, it is possible to build “zig‐zagging” **7** or helical polymers **8** with dihedral angles of ≈120° by using *meta* or pseudo‐*meta* and around 60° for *ortho* and pseudo‐*ortho* PCP (**Figure** **5**). At the same time, the chirality and functionality of the PCP remain observable. An additional benefit of using it as a side chain is the higher yield and longer chain length that result from the lower steric hindrance. Common values found are yields of up to 70% and a molecular weight of up to 10 kg mol^−1^.^[^ ^13^ ^]^

While this variability is interesting from an academic perspective, future research must still demonstrate its practical application. One of the proposed uses involves conjugated polymers, which are easily designed and synthesized due to the through‐space conjugation between rings in PCPs. Those lend themselves to semiconductor applications, especially in optoelectronics, if combined with donor and acceptor moieties. For these additional moieties, various molecules with known PCP polymers exist, such as thiophenes, carbazoles, ferrocene, benzothiadiazole, and fluorene.^[^ ^14^, ^15^ ^]^

**Figure 6:**
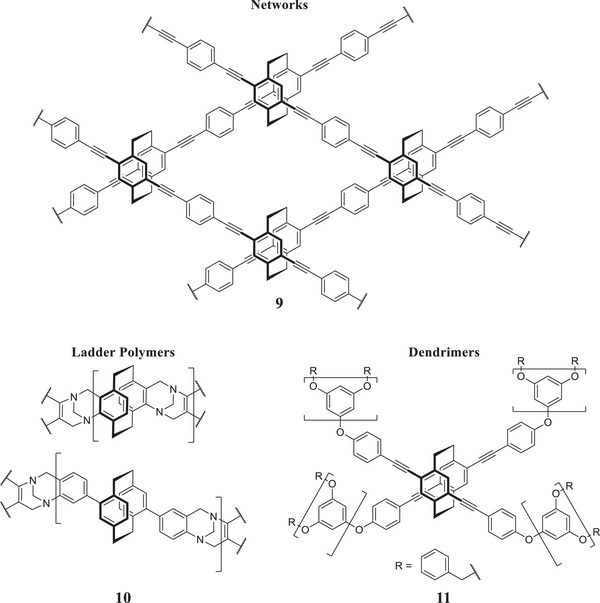
Various types of known macroscopic structures with PCP.^[^ ^16^, ^17^, ^18^ ^]^

Using PCPs with more than two substitutes also makes (chiral) networks possible, e.g. **9** (**Figure** **6**). Morisaki et al. used tetraethynyl‐PCP in combination with phenyl spacers to synthesize conjugated microporous polymers with variable surface areas.^[^ ^16^ ^]^ This makes for intriguing systems for absorption, filtering, or sensing. Again, the comparatively high cost makes the use of PCPs only viable for advanced applications, that utilize the variability and chirality, rather than as a simple storage material, for example.

While less focused on the unique properties of the PCP, it is still worth mentioning that Gon et al. also used tetrasubstituted PCP as the core of dendrimers, opening up yet another area of polymer science for PCPs (**11**, Figure 6).^[^ ^17^ ^]^ They utilized the chirality of the PCP to produce circularly polarized emitters with quantum yields of up to 0.65 and dissymmetry values of ≈2 × 10^−3^.

**Figure 7:**
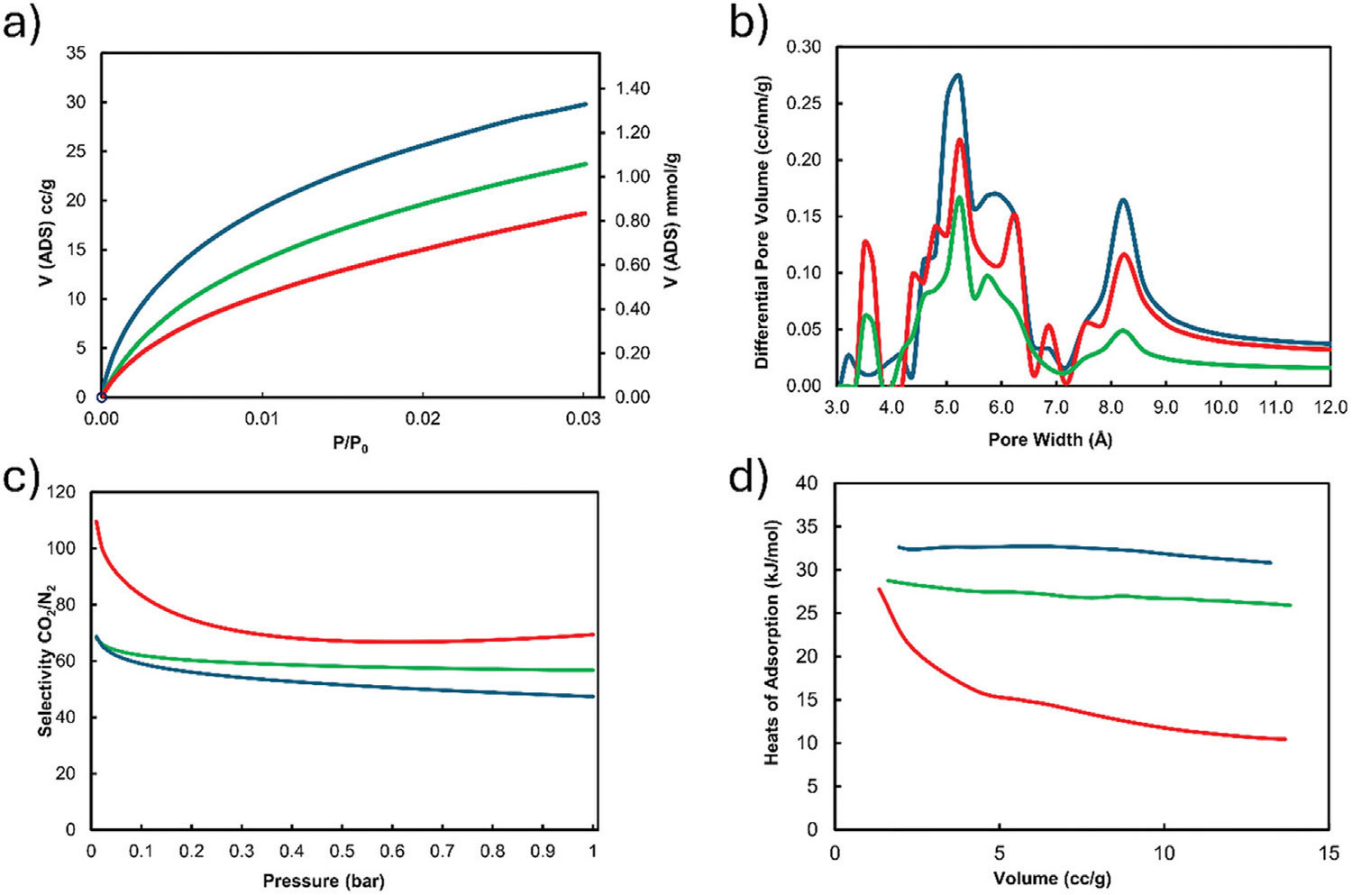
a). CO_2_ adsorption isotherms of ladder‐type PCP‐polymers measured at 273 K (desorption curves were removed for clarity); b) NLDFT pore size distribution, calculated from CO_2_ at 273K; c) IAST CO_2_/N_2_ ideal selectivity for a mixture 15/85; d) Isosteric heats of adsorption of PCP polymers. Reproduced under the terms of the CC‐BY license. 2024, published by Wiley‐VCH GmbH.^[^ ^18^ ^]^

Similarly, introducing another type of polymer, Li et al. produced ladder‐type polymers of intrinsic microporosity by employing Tröger's base formation mechanism on amine‐bearing PCPs, yielding 45–60% (**10**, Figure 6).^[^ ^18^ ^]^ The resulting insoluble polymers exhibit good surface areas and selectivity, as demonstrated by their ability to separate a CO2/N2 gas stream—a crucial property when considering technologies such as carbon capture (**Figure** **7**).

An interesting side effect of the ladder‐type structure is a stabilizing effect on the PCP. The polymers exhibit no degradation up to 430 °C, which is ≈200 °C higher than the typical degradation temperature of PCP monomers.

### 2.3 PCP in Metal‐Organic Frameworks (MOFs)

**Figure 8:**
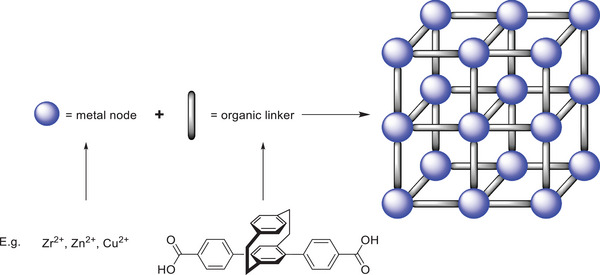
The general structure of a MOF, along with examples of building blocks.

As the final category of macroscopic structures in which PCPs can be incorporated, we aim to highlight the supramolecular scaffolding of MOFs and surface‐anchored MOFs (SURMOFs). For both structures, researchers of the KIT are at the forefront of their development, with the SURMOF even being first reported in 2007 by the KIT‐based Wöll group.^[^ ^19^ ^]^ The MOF structure comprises metal ions or clusters coordinated with organic ligands to produce highly porous crystalline materials (**Figure** **8**). These frameworks form 3D networks with exceptionally high surface areas and tunable pore sizes. MOFs are known for their versatility, finding applications in gas storage, catalysis, drug delivery, and environmental remediation.^[^ ^20^, ^21^ ^]^ Their modular design allows precise control over chemical functionality and pore geometry. SURMOFs are a subclass of MOFs grown as thin films on solid substrates. This approach combines the properties of MOFs with the advantages of surface control, enabling the development of highly ordered, layer‐by‐layer structures.

The concept of MOFs with PCP linkers is straightforward, given their rigid 3D structure, but is limited mostly to their linear *para* and pseudo‐*para* isomers. Additionally, the higher price and required synthetic efforts for PCP derivatives make them viable only for implementations that utilize their specific properties, such as chirality. Examples utilizing PCPs as organic components have been published, for instance, by Gong et al., who combined di‐ and tetrasubstituted paracyclophane carboxylic acid with zirconium.^[^ ^22^ ^]^ They used multiple PCP isomers with different in‐plane and out‐of‐plane substitutes to create five MOFs with distinct 3D topologies and structures. This, in turn, noticeably influenced their sorption behavior, underscoring the ability of PCPs to facilitate easy tunability.

Similarly, Xue et al. utilized Cu(II) as a metal node, known to be less water‐stable, in conjunction with paracyclophane dicarboxylic acid to yield a 2D MOF featuring a paddlewheel arrangement.^[^ ^23^ ^]^ The PCP stabilizes the copper coordination, making the MOF much more moisture‐resistant than comparable Cu‐MOFs. While other Cu‐MOFs decompose in water within minutes, the incorporation of PCP enables their MOF to survive for years, as long as the pH remains between 3 and 8. This allows their use in water harvesting, storage, and separation, making them candidates for catalytic processes in aqueous media.

As discussed earlier, PCPs also enable the incorporation of chirality into these macroscopic structures. Jiang et al. and Cakici et al. used PCP linkers to produce optically active MOFs and SURMOFs.^[^ ^24^, ^25^ ^]^ Jiang utilized their framework with Zr to yield a circularly polarized luminescence emitter with a significantly enhanced dissymmetry factor and luminescence quantum yield (8.3 × 10^−3^ and 0.87 respectively), thereby suppressing quenching effects through the rigid structure. Cakici utilized the chiral PCP linker with Zn nodes to synthesize a SURMOF, aiming for enantioselective separation in the gas phase. They could successfully demonstrate this by showing a preferential uptake of R‐limonene into the lattice while *S*‐limonene remained outside.

## 3 PCP in Chemical Vapor Deposition (CVD)

**Figure 9:**
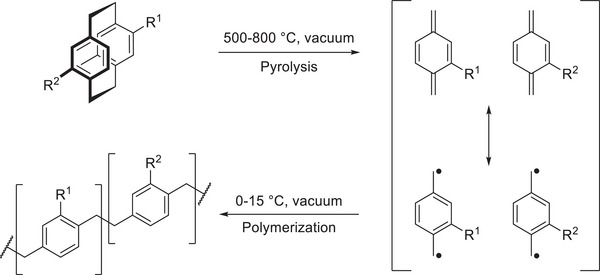
CVD process from functionalized PCP to parylene.

A different approach to polymerizing PCP utilizes its structure as a driving force rather than as a building block. The ring strain of the PCP (130 kJ mol^−1^)^[^ ^26^ ^]^ enables its use in CVD, producing functionalized poly(*para*‐xylenes), commonly known as parylenes, which are optimal for coating various surfaces. During the CVD process, PCP undergoes pyrolysis at elevated temperatures, typically around 500–800 °C, breaking into reactive diradical intermediates known as quinodimethanes (**Figure** **9**, right). These intermediates polymerize upon condensation on cooler substrate surfaces, forming uniform, pinhole‐free parylene coatings. These films are highly valued for their excellent dielectric properties, chemical resistance, and biocompatibility, making PCP‐based CVD a potential key technique in electronics, medical device encapsulation, and barrier coatings.^[^ ^27^ ^]^ The rigid and controlled structure of PCP ensures high purity and efficiency in this process.

**Figure 10:**
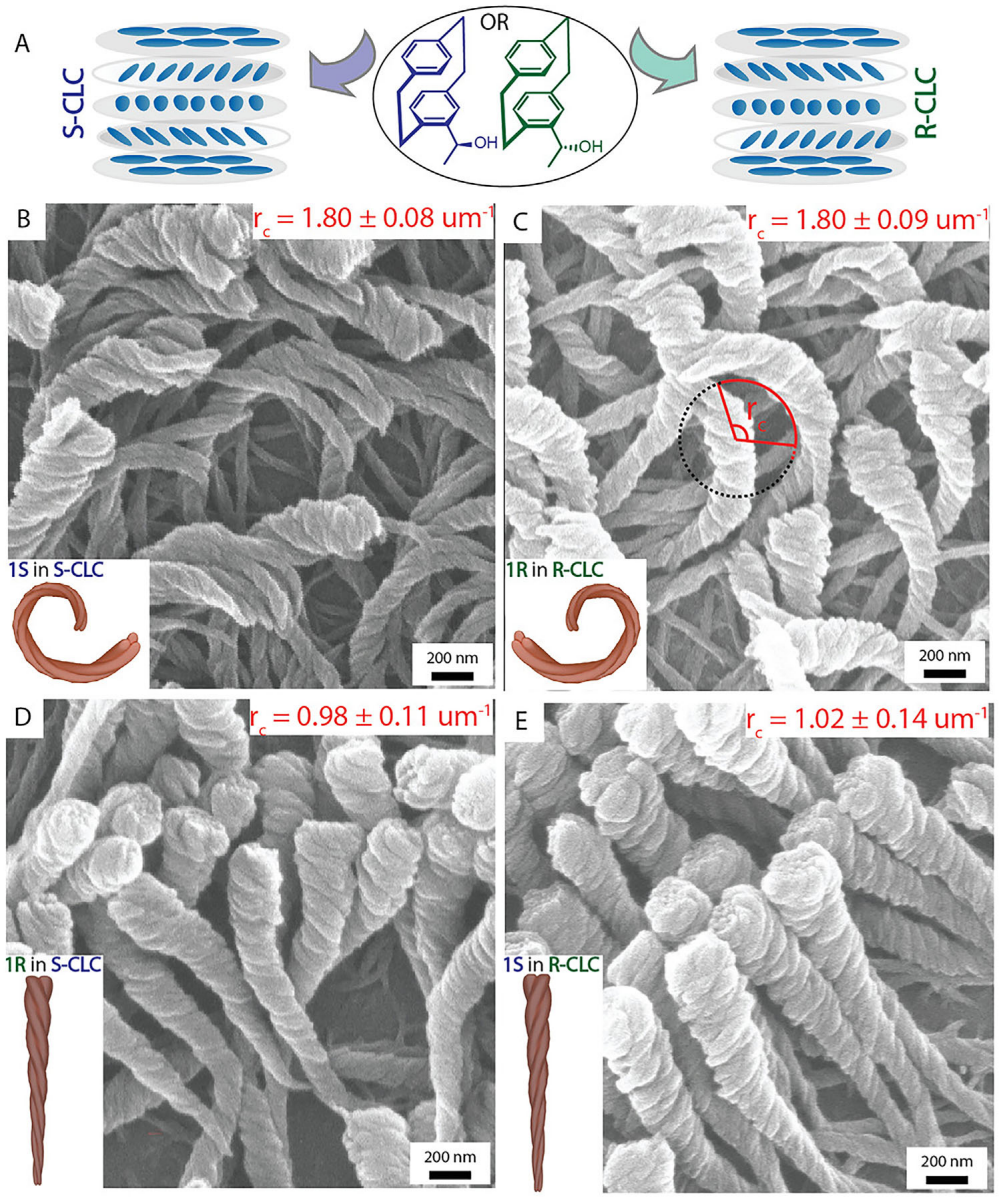
Nanohelices are produced on CVD‐coated surfaces. Reproduced with permission.^[^ ^37^ ^]^ 2021, Wiley‐VCH GmbH.

Additional groups can be introduced via the PCP monomer to give a functionalized surface. This process sacrifices the properties of PCP, such as its intrinsic chirality, in exchange for high solvent resistance even at high temperatures, a high melting point, a low dielectric constant, and very good barrier properties.^[^ ^28^ ^]^ The scalability of the CVD process, combined with the absence of alternatives that produce parylenes as a side product and in a homogeneous form, makes this the PCP application with the greatest promise for industrial applications. It is currently mainly found in fields such as biointerfaces or resistant coatings in electronic devices.^[^ ^29^, ^30^ ^]^ The parylenes can be outfitted with nearly all common functional groups, providing a very broad range of possible options for surface functionality. To provide some examples, Bichlmeier et al. utilized hydrosilane PCP to produce a layer of parylenes with high transparency, biocompatibility, and excellent plasma etch resistance, making it an ideal material for coating medical electronics.^[^ ^31^ ^]^ The extreme stability and tunable functionality of the parylenes even led to applications in highly specialized fields, such as aerospace engineering, where Fundeanu et al. utilized amino‐PCP to develop a highly resistant coating that simultaneously enhanced adhesive strength.^[^ ^32^ ^]^ The introduced groups can also be further reacted in post‐modifications via, for example, click or cross‐coupling chemistry or by grafting functional molecules onto the uniform parylene film of controllable thickness.^[^ ^33^, ^34^ ^]^ One major use of this is the immobilization of various biomolecules on various surfaces, such as steel, polyethylene, or glass.^[^ ^35^, ^36^ ^]^ Another use is the bottom‐up transfer of properties, which was demonstrated, for example, by Varadharajan et al., who used a coating of parylenes, both achiral and chiral, to influence the contour length, pitch, and twist of nanohelices grown on CVD‐coated surfaces (**Figure** **10**).^[^ ^37^ ^]^ Through minute changes in the composition of used PCPs, a wide array of structures was possible, an effect known mostly from biological systems.

These examples provide a small but meaningful insight into the vast possibilities of PCP‐based coatings, particularly considering the numerous potential post‐modifications.

## 4 Conclusion

As the KIT celebrates 200 years of scientific innovation, [2.2]paracyclophane is one field that demonstrates the institution's contributions to cutting‐edge material science. [2.2]Paracyclophane continues to demonstrate its potential as a cornerstone in advanced polymer development, offering properties such as a rigid 3D structure, chirality, and through‐space conjugation. The versatility of its derivatives has enabled applications ranging from gas separation and chiral sensing to optoelectronics and surface engineering. Furthermore, its integration into MOFs and SURMOFs highlights its relevance in emerging fields for developing tunable and highly functional materials. At the same time, many challenges and questions surrounding the use of PCPs in macroscopic structures are still open and need to be investigated. For one, the price remains high with no cost‐effective, targeted process available. Instead, PCP remains a byproduct of xylene pyrolysis or a subject of multi‐step synthesis on a laboratory scale. This is doubly true for enantiomerically pure molecules. The first steps toward a purposeful synthesis were made for selected derivates, but to utilize the potential of PCPs on a commercial level, more work is needed.^[^ ^38^, ^39^ ^]^ At the same time, more fundamental research on PCP monomers is needed to broaden the scope of achievable polymers and to better understand the involved reaction mechanisms in polymerizations, thereby achieving more homogeneous materials. With this, PCP‐based macrostructures can start to better compete with already established systems, similar to what its small molecules achieved before. In the meantime, focusing on the areas where PCP offers unique benefits, such as the synthesis of parylenes through CVD, can outweigh the cost and scalability drawbacks.

We believe that more research, not only at KIT, should focus on enhancing its synthetic accessibility and exploring novel applications to benefit from its continued role as an innovative component in material science innovation.

## Conflict of Interest

The authors declare no conflict of interest.
